# Awareness and knowledge of developmental co-ordination disorder among physicians, teachers and parents

**DOI:** 10.1111/j.1365-2214.2012.01403.x

**Published:** 2012-07-24

**Authors:** B N Wilson, K Neil, P H Kamps, S Babcock

**Affiliations:** * Alberta Health Services, Alberta Children's Hospital; † Vice President Research, Vision Critical; ‡ Private Practice: KAMPS (Kinesiology and Meta-cognitive Psychological Services); 4 Regional School Health, Alberta Health ServicesCalgary, AB, Canada

**Keywords:** awareness, DCD, developmental co-ordination disorder, diagnosis, knowledge

## Abstract

**Background** Obtaining a diagnosis of developmental co-ordination disorder (DCD) is a long, inconsistent and frustrating journey for families, with apparently little awareness of DCD in schools or the medical community.

**Methods** An online survey was completed by 1297 participants: parents (*n* = 501), teachers (*n* = 202), family/general physicians (*n* = 339) and paediatricians (*n* = 255).

**Results** Only 20% of the sample had knowledge of DCD, with 41% of the paediatricians and 23% of family/general physicians familiar. Of participants who have awareness, only 11–59% have knowledge of the impact of DCD on social, emotional and physical health. Less than 30% of physicians have awareness of the secondary consequences. Few physicians diagnose DCD and less than one-third believe it is easy to make a diagnosis; this is in contrast to the fact that most parents report confidence in their physician's ability to make a timely diagnosis.

**Conclusions** If less than one-half of physicians have knowledge of DCD and even fewer are knowledgeable of the secondary consequences of the condition, it is not surprising that DCD is infrequently diagnosed and that families need to search for support. This survey confirms observations that the condition is not well known and there is a need for greater awareness of DCD.

## Introduction

Although the childhood impact (Sugden 2006) and long-term sequelae (Cantell *et al*. 2003, 2008) of developmental co-ordination disorder (DCD) is well known, it remains a relatively unrecognized and underfunded condition. Families who suspect their child has DCD typically spend months and sometimes years seeking medical and educational advice to identify the problem and obtain intervention and support (Rodgers & Mandich, 2005; Missiuna *et al*. 2006a,b). The diagnostic pathway for DCD can involve a dozen tests (some repetitive) and eight or more health care professionals. Although research done retrospectively with families who eventually obtained a diagnosis suggests a long and frustrating journey with little awareness of DCD in schools or the medical community, only Kirby and colleagues (2007) have studied the knowledge of DCD in a medical professional group in the UK. They found that 67.3% of child and adolescent psychiatrists who regularly assess children for attention deficit hyperactivity disorder rated their knowledge of motor co-ordination difficulties of their clients as ‘poor’ or ‘very poor’, compared with 13.7% of paediatricians having little knowledge. Although most of these physicians asked clients and families about motor problems, few knew the term ‘DCD’ or its definition, and the majority requested more training.

As a first step in raising awareness of DCD as a significant developmental condition with impact into adolescence and adulthood, the aim of this study was to measure current awareness and knowledge of DCD among key stakeholder groups.

## Methods

The study was conducted as a short, online survey. Four groups of participants were recruited: parents of children between 3 and 12 years of age (*n* = 501), teachers of children between 5 and 12 years (*n* = 202), family/general physicians working in the community, whose practice was comprised of least 15% children (*n* = 339), and paediatricians (*n* = 255). The data were collected between 10 December 2010 and 28 February 2011. The response rate was 29%.

Parent and teacher sample was drawn from Angus Reid Forum, a national market research and public opinion panel. The survey was deployed via email invitation to a demographically balanced sample (excluding Quebec) of English-speaking Canadians. The physician sample was recruited from Canada, the USA and UK (50%, 42% and 8%, respectively) through specialized, online medical research panels. A broader geographic range was targeted for physicians in order to achieve a maximum sample size, as physicians were a priority sample group in the study.

The entire sample (1297 participants) was initially asked to identify their familiarity with 17 common childhood conditions. Presented in random order, the list included DCD and similar terms including Dypraxia, Motor Learning Disability and Clumsy Child Syndrome. A 5-point Likert scale was used to rate their level of familiarity; ‘very familiar’, ‘somewhat familiar’, ‘somewhat unfamiliar’, ‘very unfamiliar’ and ‘I have not heard of this condition at all’.

## Results

Table 1 shows that, although 83–87% of all groups participants are ‘very familiar’ or ‘somewhat familiar’ with attention deficit hyperactivity disorder, learning disability and obsessive compulsive disorder, only 20% are familiar with DCD. When broadening the description of other terms describing motor co-ordination disorders (dyspraxia, motor learning disability, and clumsy child syndrome), DCD still remains one of the least known among the childhood conditions commonly diagnosed during the target age range.

**Table 1:** Percentage of respondents who were ‘very familiar’ or ‘somewhat familiar’ with conditions

	Total (%)	Family doctor (%)	Paediatrician (%)	School teacher (%)	Parent (%)
*n* = 1299	*n* = 339	*n* = 255	*n* = 202	*n* = 501
Attention deficit hyperactivity disorder	87	93	99	96	74
Learning disability	83	78	93	97	75
Obsessive compulsive disorder	83	91	91	85	73
Autism	81	78	99	92	69
Dyslexia	77	70	85	88	74
Mental retardation	77	82	96	81	62
Spina bifida	65	74	95	60	46
Asperger's syndrome	65	66	94	86	40
Autism spectrum disorder	64	70	98	78	38
Generalized developmental delay	57	66	95	66	27
Oppositional defiance disorder	53	63	85	76	20
Chromosomal disorders	50	58	93	44	25
Conduct disorder	47	64	82	57	14
Motor learning disability	39	34	55	60	27
Dyspraxia	27	33	61	21	7
Developmental co-ordination disorder	20	22	41	23	6
Clumsy child syndrome	16	17	35	14	6

Of the paediatricians, 15% are ‘very familiar’ and 26% ‘somewhat familiar’ with the term DCD, totalling 41% classified as ‘familiar’. Of family/general physicians, 4% are ‘very familiar’ and 19% ‘somewhat familiar’ with DCD, for a total of 23% ‘familiar’.

We then asked questions specific to DCD to the subgroup of teachers, and family/general physicians and paediatricians who stated they were ‘very’ or ‘somewhat familiar’ with DCD and/or the three other developmental motor terms (*n* = 46 and 179, respectively). Table 2 shows the percentages of professionals who state that certain features are ‘part of the condition of DCD’. Although 70–79% are aware of the motor components of the disorder, only 11–59% are aware of the social, emotional and physical health aspects of the condition.

**Table 2:** Percentages of professionals who state that certain features were ‘part of the condition of DCD’

	Physicians (%)	Teachers (%)
Common motor features of DCD		
Motor learning difficulties	79	74
Difficulty printing and/or writing	77	72
Gross motor and fine motor skills delay	70	74
Non-motor common features of DCD		
Low self-esteem	59	41
Poor physical fitness	49	43
Sensory processing challenges	42	30
Anxiety	40	30
Difficulty making friends	40	33
Poor social skills	37	22
Depression	28	11
Less common features		
Poor academic performance	41	28
Average (or above average) cognitive ability	48	30
Below average cognitive ability	16	11
Higher than average risk for suicide	20	7
Obesity	17	9

DCD, developmental co-ordination disorder.

Although physicians are slightly more aware of common features of DCD than teachers, their awareness seems to be in contrast to the number who identify children in their practice who are ‘self-conscious and stressed about his or her physical skills’, ‘have trouble keeping up in sports or writing and drawing’ or ‘who are clumsy and accident-prone‘ (Table 3). Given the prevalence of DCD, the number of children recognized by physicians as having motor problems also appears very low.

**Table 3:** Mean number of children seen in physicians’ practice with features of developmental disorders

	Paediatricians	Family/general physicians
Has evidence of difficulty learning (learning disability)	91	17
Demonstrates oppositional behaviour	53	11
Is self-conscious and stressed about his or her physical skills	32	9
Has trouble ‘keeping up’ with other kids in physical games or sports	44	9
Cannot complete tasks such as writing, drawing or handling small objects, in a way that's adequate for their age	44	8
Appears to be clumsy and accident prone when compared to their peers	28	6
Has difficulty moving their mouth in such a way that their speech is easy to understand, yet passes standard speech tests	12	3

Physicians were also asked ‘Have you diagnosed a child with DCD?’ Only 23% of paediatricians and 9% of family doctors have diagnosed DCD. Three-quarters were surprised at the incidence of DCD, and 94% and 89% (paediatricians and family doctors, respectively) want more education on DCD and see the need for more research. Only one-quarter of paediatricians and one-third of family doctors believe DCD is easy to diagnosis; this is in sharp contrast to the fact that over 70% of parents are confident that their physician would be able to make an accurate and timely diagnosis ‘if my child had a specific condition’.

Finally, all participants were asked about services needed for children with developmental needs. An exceptionally high percentage agree that there is a need for more education about children with special needs: over 90% of physicians want more education about DCD, and over 85% believe there are significant benefits to making an accurate and early diagnosis. Over 80% of parents also feel that there should be more education for parents about the signs of DCD and other childhood conditions. Ninety-seven per cent of teachers believe that accurate diagnoses are critical for educators to know how to help children with special needs and 85% feel the education system would currently not be able to adequately support children with DCD due to the lack of awareness and knowledge.

## Discussion

If only 23–41% of physicians are familiar with DCD and, of those who are aware, only 11–59% are knowledgeable about the psychological and secondary consequences of the condition, it is not surprising that the disorder is infrequently diagnosed and that families need to search for a diagnosis and for support. This survey confirms observations by clinicians and researchers that DCD is not well known and that many children are likely to be ‘missed’ or to be misdiagnosed. Less than one-quarter of physicians surveyed have diagnosed DCD and 70% do not feel that a diagnosis is easy. The need for greater awareness of DCD and education in the general public, and with physicians and teachers specifically, is evident.

Increasing awareness of DCD requires effective translation of our knowledge about DCD – prevalence, impact on daily life, and the serious consequences if left unrecognized and unsupported. Any approach adopted to increase recognition of DCD must be in accordance with the context of the region and match the target group, whether this is the general public, educational professionals or health practitioners. Researchers have an important role to play by providing written and oral reports which are user-friendly and accessible to parents and service providers, as well as presented in scientific journals and at large conferences (Rosenbaum 2012). Parents are the key group to advocate for early identification and better services in health and education, and professionals can support and empower parents to connect with policy makers, be aware of the current evidence, and take affirmative action.

Two initiatives which are increasing awareness of DCD can be used as exemplars. A recent one is that of German-speaking countries who, with the European Academy of Childhood Disabilities, initiated a consensus process with international participation which resulted in European Academy of Childhood Disabilities Recommendations (and German-Swiss Clinical Practice Guidelines) for healthcare providers (Blank *et al*. 2012). As well as influencing multidisciplinary best practice, the authors intend that the publication will raise community awareness and increase professional attention to DCD in Europe. The Recommendations are currently being adapted for use in the UK by a multidisciplinary group; adoption and adaptation of these practice guidelines in other countries will impact recognition of DCD in children. The second example comes from Canada where there are neither provincial nor national guidelines. A comprehensive knowledge translation approach has been utilized by the *CanChild* group to increase recognition by physicians (Missiuna *et al*. 2008). Numerous booklets and pamphlets were made and widely accessed by parents and professionals from their website (http://www.canchild.ca). Educational outreach programmes and other innovative practices designed to increase physicians’ ability to accurately identify DCD were developed (Missiuna *et al*. 2006c).

As more professionals acquire greater awareness of DCD, more consistent processes for screening and assessment can be developed across the various contexts of different regions. Obtaining parent and/or teacher questionnaires is an important starting point, providing an opening for further interview with the parents on how their child's motor challenges are impacting daily activities. Only when questionnaire and interview results point to significant motor problems is assessment with a standardized instrument warranted. Rather than beginning with a test battery and multiple professional visits, a ‘staged assessment procedure’ is both economical and less burdensome to the child, family and service system.

## Key messages

1. DCD was the least well known of 17 common conditions among parents, teachers and physicians, even when other terminology was included.
2. Less than 33% of general physicians and only 41% of paediatricians are familiar with DCD.
3. Although 70% of physicians and teachers identified the common *physical* characteristics of DCD, less than 30% identified the *psychological and secondary consequences* of DCD, including low self-esteem, poor fitness, anxiety and depression.
4. Only 23% of paediatricians and 9% of general physicians have diagnosed DCD.
5. Although 70% of parents are confident that their child's physician would accurately and quickly diagnosis a specific condition, only 30% of physicians believe that diagnosing DCD would be relatively easy.
